# Intrinsic properties and neuropharmacology of midline paraventricular thalamic nucleus neurons

**DOI:** 10.3389/fnbeh.2014.00132

**Published:** 2014-04-17

**Authors:** Miloslav Kolaj, Li Zhang, Michael L. H. J. Hermes, Leo P. Renaud

**Affiliations:** Neuroscience Program and Department of Medicine, Ottawa Hospital Research Institute, University of OttawaOttawa, ON, Canada

**Keywords:** midline thalamic nuclei, electrophysiology, peptides, diurnal and seasonal changes, burst firing

## Abstract

Neurons in the midline and intralaminar thalamic nuclei are components of an interconnected brainstem, limbic and prefrontal cortex neural network that is engaged during arousal, vigilance, motivated and addictive behaviors, and stress. To better understand the cellular mechanisms underlying these functions, here we review some of the recently characterized electrophysiological and neuropharmacological properties of neurons in the paraventricular thalamic nucleus (PVT), derived from whole cell patch clamp recordings in acute rat brain slice preparations. PVT neurons display firing patterns and ionic conductances (I_T_ and I_H_) that exhibit significant diurnal change. Their resting membrane potential (RMP) is maintained by various ionic conductances that include inward rectifier (Kir), hyperpolarization-activated nonselective cation (HCN) and TWIK-related acid sensitive (TASK) K^+^ channels. Firing patterns are regulated by high voltage-activated (HVA) and low voltage-activated (LVA) Ca^2+^ conductances. Moreover, transient receptor potential (TRP)-like nonselective cation channels together with Ca^2+^- and Na^+^-activated K^+^ conductances (K_Ca_; K_Na_) contribute to unique slow afterhyperpolarizing potentials (sAHPs) that are generally not detectable in lateral thalamic or reticular thalamic nucleus neurons. The excitability of PVT neurons is also modulated by activation of neurotransmitter receptors associated with afferent pathways to PVT and other thalamic midline nuclei. We report on receptor-mediated actions of GABA, glutamate, monoamines and several neuropeptides: arginine vasopressin, gastrin-releasing peptide, thyrotropin releasing hormone and the orexins (hypocretins). This review represents an initial survey of intrinsic and transmitter-sensitive ionic conductances that are deemed to be unique to this population of midline thalamic neurons, information that is fundamental to an appreciation of the role these thalamic neurons may play in normal central nervous system (CNS) physiology and in CNS disorders that involve the dorsomedial thalamus.

## Introduction

The thalamus, the principal gateway for information from various sensory modalities to reach higher cognitive centers, is a critical component of conscious behavior. The midline and intralaminar nuclei, initially regarded as relays in a “nonspecific” thalamocortical arousing system, have now gained recognition not only for their specific two way connectivity with areas of cortex and striatum, but also for their involvement in a variety of distinct functions, notably vigilance and arousal, nociception, stress, memory and cognition, and motivated behaviors (Groenewegen and Berendse, 1994; Bhatnagar et al., 2002; Van der Werf et al., 2002; Sewards and Sewards, 2003). In addition, there is evidence that dysfunction of this region of thalamus may contribute to and/or cause various psychopathologies, sleep disorders and limbic epilepsy (see reviews by Benarroch, 2008; Rajasekaran et al., 2009; Price and Drevets, 2010). Knowledge of the intrinsic and synaptic properties that regulate the excitability of neurons is fundamental to understanding neural communication, integration and information processing in the central nervous system (CNS). To date, interest in thalamic function at the cellular level has largely focused on the role of neurons in specific relay and reticular nuclei, whereas comparatively little research has been devoted to the cellular physiology and function of neurons constituting the midline and intralaminar thalamic nuclei. As a contribution towards this objective we focus this review on the electrophysiology and pharmacology of neurons in paraventricular thalamic nucleus (PVT), a stable midline structure throughout mammalian evolution. In the rat, PVT extends rostrocaudally below the third ventricle and is the most dorsal component of the thalamic midline-intralaminar nuclear complex. In both rodents and primates (monkey), PVT neurons have extensive connectivity with a variety of neurons in the hypothalamus, brainstem, limbic regions (bed nucleus of stria terminalis, nucleus accumbens, amygdala) and prefrontal cortex (Berendse and Groenewegen, 1990; Su and Bentivoglio, 1990; Moga et al., 1995; Otake and Ruggerio, 1995; Kawano et al., 2001; Peng and Bentivoglio, 2004; Li and Kirouac, 2008, 2012; Vertes and Hoover, 2008; Hsu and Price, 2009). The observation that PVT neurons exhibit enhanced immediate early gene (e.g., *c-Fos*) expression during arousal and after exposure to various stressors (e.g., restricted mobility, food deprivation) and psychostimulants (reviewed in Price and Drevets, 2010) suggests that these neurons participate in a variety of behaviors. PVT also appears as a common nodal structure in central pathways integrating salt appetite, energy balance and food reward (Kelley et al., 2005; Parsons et al., 2006; Shekhtman et al., 2007; Kampe et al., 2009). Collectively, these findings implicate an involvement of this midline cell group in survival-oriented behaviors, homeostasis and possibly addictions.

## Intrinsic properties

PVT neurons have somata with diameters in the 12–20 micron range and 3–7 aspiny main dendrites that extend for several hundred microns (Richter et al., 2006; Zhang et al., 2009, 2010), and synthesize glutamate for rapid neurotransmission (Christie et al., 1987; Csáki et al., 2000; Hur and Zaborszky, 2005). Whereas PVT has been assigned anterior and posterior components based on functional considerations (e.g., see Bhatnagar et al., 2002) and differing anatomical connectivity (e.g., Li and Kirouac, 2012), the morphology of PVT neurons based on intracellular labeling reveals relatively small variations along the rostrocaudal axis of the nucleus (Brunton and Charpak, 1998; Heilbronner and Flügge, 2005). Information presented here on their intrinsic electrical properties and synaptic pharmacology is derived from observations obtained with patch-clamp recording techniques in rat brain slice preparations. In most earlier reports, data were acquired from slices prepared during the animal’s resting period, e.g., at zeitgeber (ZT) 4–10, (ZT 0 is light on; animals maintained on a 12 h light/12 h dark cycle). However, PVT neurons are known to receive an innervation from the suprachiasmatic nucleus (Peng and Bentivoglio, 2004; also see below) which contains the master circadian pacemaker (Reppert and Weaver, 2001), and to express diurnal changes in early gene expression (Peng et al., 1995; Novak and Nunez, 1998). These features, together with the fact that rats are nocturnal feeders prompted a comparison of intrinsic properties in neurons recorded in slices prepared during ZT 2–6 (day period) with those in slices prepared during ZT 14–18 (night period), when animals are active (Kolaj et al., 2012). Where data are available, these comparisons are considered in the following review.

### Membrane properties and activity patterns

**Figure 1:**
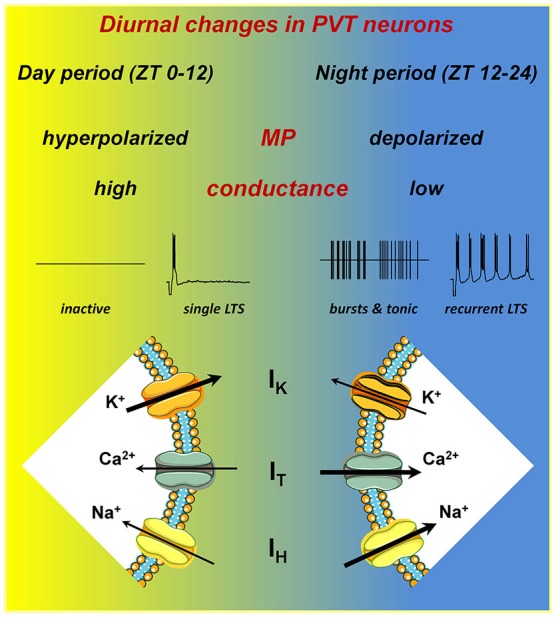
**PVT neurons express diurnal changes**. A schematic illustrating the differences in intrinsic electrical properties of PVT neurons recorded in slices from different ZT periods. In night period slices, neurons have a more depolarized resting membrane potential (RMP), lower resting membrane conductance, in part owing to lower overall K^+^ currents (I_K_), and larger amplitude T-type Ca^2+^ (I_T_) and hyperpolarization-activated cation (I_H_) currents. These changes result in increased spontaneous tonic and burst firing, and enhanced recurrent activity subsequent to generation of a low-threshold spike (LTS). For details, see Kolaj et al. (2012).

Compared with neurons in day period slices, neurons in night period slices have a mean resting membrane potential (RMP) that is ∼10 mV more depolarized and have a significantly lower membrane conductance (Figure 1). A comparison of the mean current-voltage relationship of neurons from day period and night period slices revealed a differential membrane current with a reversal potential near the K^+^ equilibrium potential, implying that one or more K^+^ conductances are contributing to the observed difference in membrane potential (MP; Kolaj et al., 2012). In addition, both cell attached and whole cell recordings reveal that most PVT neurons in day period slices are silent, contrasting with neurons in night period slices where the majority of PVT neurons display tonic or burst firing (Figure 1).

### Low-threshold Ca^2+^ spike, T-type Ca^2+^ channels and I_T_

Similar to thalamocortical and reticular thalamic neurons, PVT neurons exhibit two modes of activity, tonic and burst firing (reviewed in McCormick and Bal, 1997). Thus, a step depolarization from RMP initiates tonic firing with varying degrees of spike frequency adaptation, whereas a step depolarization from a more hyperpolarized MP or the offset of a step hyperpolarization from RMP triggers a low-threshold Ca^2+^ spike, or LTS (Figure 1, single LTS). The LTS is often sufficient to elicit a burst of tetrodotoxin (TTX)-sensitive Na^+^ action potentials, and may be followed by a slow afterhyperpolarization (sAHP) and a series of oscillatory bursts (Figure 1, recurrent LTS). The LTS results from activation of low voltage-activated (LVA) T-type Ca^2+^ channels, which are major contributors to rhythmic oscillatory behavior in thalamic and other neurons (reviewed in Perez-Reyes, 2003) and can be blocked by nickel (Ni^2+^) (Huguenard, 1996). In PVT neurons, Ni^2+^ can be seen to completely arrest spontaneous bursting or LTS-induced recurring bursting activity, whereas cadmium (Cd^2+^) has no influence on these activities (Zhang et al., 2009), observations implying that HVA Ca^2+^ channels have little or no contribution to bursting behavior in PVT neurons, although they are important for tonic firing (see Wong et al., 2013).

The mammalian genome encodes genes for 3 distinct isoforms of the T-type Ca^2+^ channel, Ca_v_3.1 (or α_1G_), Ca_v_3.2 (or α_1H_), and Ca_v_3.3 (or α_1I_) (reviewed in Perez-Reyes, 2003). Our RT-PCR analysis in rat brain revealed that the major subunit in PVT is Ca_v_3.1, with fewer Ca_v_3.3 and almost no Ca_v_3.2 (Kolaj et al., 2012). This is consistent with earlier investigations reporting that the Ca_v_3.1 subtype is most dominant in midline thalamus in rat (Talley et al., 1999; McKay et al., 2006). However, a recent study in mice featuring the importance of the Ca_v_3.2 subtype in hyperalgesia (Chen et al., 2010) suggests a possible variation in subtypes between rodent species.

**Figure 2:**
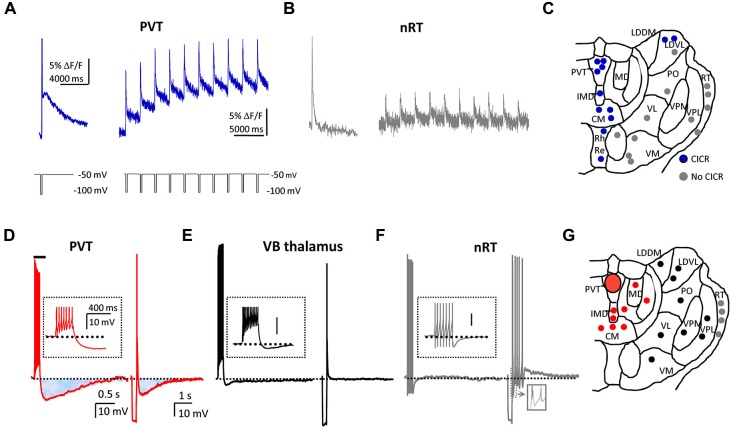
**PVT neurons express unique T-type Ca^2+^ channel-mediated intracellular calcium profiles, and sAHPs. (A)** On the left, a typical Ca^2+^ response profile in a single PVT neuron displays a fast and a slow phase of the Ca^2+^ signal in response to a single voltage pulse that activates T-type Ca^2+^ channels. On the right, average trace from 12 neurons that exhibit Ca^2+^ response profiles consistent with calcium-induced calcium release (CICR) in response to repetitive activation of T-type Ca^2+^ channels. **(B)** For contrast, trace from a single nRT neuron (left) exhibits no slow Ca^2+^ response and average trace from 13 neurons (right) shows no evidence of CICR. For details, see Richter et al. (2005). **(C)** Distribution of thalamic neurons that did (blue circles) or did not (gray circles) exhibit CICR in response to repetitive activation of T-type Ca^2+^ channels. Abbreviations and scheme are based on the rat atlas by Paxinos and Watson (1998) (Reproduced in part with permission from Richter et al., 2005). **(D)** Voltage traces from the same PVT neuron illustrate a spike train-induced (left) and an LTS-induced (right) sAHP (shaded areas). **(E, F)** Using similar protocols, representative traces indicate that similar sAHPs are not observed in ventrobasal (VB; black trace and symbols) or reticular thalamic (nRT; gray trace and symbols) neurons. **(G)** Distribution of tested neurons to depict that only cells in midline and intralaminar thalamus displayed sAHPs (red circles). Abbreviations and scheme are based on the rat atlas by Paxinos and Watson (1998) (Reproduced in part with permission from Zhang et al., 2010).

A further evaluation of properties of LVA Ca^2+^ channels with confocal microscopy and Ca^2+^ imaging techniques reveals features that may be unique to LVA T-type Ca^2+^ channels in PVT neurons. First, LVA Ca^2+^ channels are strongly represented in both somata and dendrites, but the magnitude of LVA Ca^2+^ channel-evoked Ca^2+^ transients is significantly greater in proximal dendrites (up to 20 µm from the soma) than in somata. Also, these Ca^2+^ channels display considerable heterogeneity in their distribution (or lack thereof) within different dendrites (see Richter et al., 2006). Second, Ca^2+^ entry via LVA Ca^2+^ channels is coupled to Ca^2+^-induced Ca^2+^ release (CICR) from intracellular Ca^2+^ stores, a phenomenon not apparent in reticular thalamic nucleus or ventrolateral thalamocortical relay neurons (Figures 2A–C; see Richter et al., 2005). It is anticipated that further investigations will reveal a link between CICR and the slow afterpotentials (Figures 2D–G; see below) that also appear to be uniquely featured in midline thalamic neurons.

#### I_T_: day period vs. night period

The increase in burst firing activity observed in PVT neurons in night period slices (Figure 1) coincides with an increase in I_T_ amplitude at MPs more depolarized than −60 mV (Kolaj et al., 2012). Although an underlying mechanism remains to be identified, RT-PCR analysis in tissue samples from anterior PVT reveals a significant increase in mRNA for both Ca_v_3.1 and Ca_v_3.3 isoforms in night period slices (Kolaj et al., 2012). In thalamocortical and reticular thalamic neurons, an increased “window” component of I_T_ originating from the region of overlap between steady-state activation and inactivation curves of I_T_ has been proposed to contribute to slow rhythmic burst firing (Crunelli et al., 2005; Blethyn et al., 2006). Thus a “window” I_T_ that is increased in PVT neurons in night period slices could permit amplification of bursting behavior at more depolarized RMPs.

### Hyperpolarization-activated nonselective cation channels (HCN) and I_H_

The majority of PVT neurons display a depolarizing voltage sag during a sustained membrane hyperpolarization and a slow inward current relaxation in response to a hyperpolarizing voltage step (see Figure 2 in Zhang et al., 2006b), features consistent with a membrane current (I_H_) resulting from activation of HCN channels (reviewed in Biel et al., 2009). In thalamocortical neurons, HCN channels contribute to both RMPs and to oscillatory and bursting activity (e.g., McCormick and Pape, 1990). Consistent with a similar role in PVT neurons is the observation of a gradual membrane hyperpolarization and cessation of spontaneous oscillatory behavior following bath application of ZD7288, a selective inhibitor of HCN channels (see Figure 4A in Kolaj et al., 2012). HCN channels are composed of four members, HCN 1–4, with a wide CNS expression (Monteggia et al., 2000; Notomi and Shigemoto, 2004; Biel et al., 2009). A recent RT-PCR analysis indicates that the major subunit in midline thalamus is HCN2 followed by HCN4 > HCN3 > HCN1 (Kolaj et al., 2012).

#### I_H_: day period vs. night period

A comparison of the effect of ZD7288 on neurons maintained at similar MPs (−60 mV) revealed a significantly larger hyperpolarization in neurons from night period slices compared to neurons from day period slices (Kolaj et al., 2012). A voltage clamp assessment of I_H_ indicated an increase in I_H_ in neurons from night period slices, starting at holding potentials more hyperpolarized than −80 mV (Kolaj et al., 2012). However, RT-PCR assessment of mRNA expression of individual HCN channel isoforms in punch biopsies from the anterior PVT area revealed no significant difference between day and night period slices (Kolaj et al., 2012), suggesting that diurnal changes in I_H_ result from a mechanism that is different from that underlying changes in I_T_.

### Enhanced spontaneous and induced firing patterns in night period slices

In thalamic neurons, burst and tonic firing patterns are closely related to MPs, and the data reviewed here indicate a decrease in resting K^+^ conductance as well as an increase in T-type Ca^2+^ and HCN-type of conductances in PVT neurons in night period slices (Figure 1). These changes influence spontaneous and induced firing behaviors. As reported in Kolaj et al. (2012), no marked differences exist in the response of neurons from day vs. night period slices to step changes in MP to −50 mV, where most neurons display tonic firing. However, upon a −10 mV change in MP to −60 mV, most neurons in day period slices became silent while most neurons from night period slices exhibited LTS-induced burst firing. In addition, upon return of MP to ~ −60 mV following a step hyperpolarization of sufficient duration, most PVT neurons in day period slices responded with a single LTS, while a similar protocol applied to PVT neurons in night period slices triggered recurring LTS-initiated bursts (Figure 1).

### Inward rectifier K^+^ channels (Kir)

Current-voltage relationships in PVT neurons typically demonstrate a strong time independent inward rectification (see Figure 2 in Zhang et al., 2006b), a feature indicative of inward rectifier K^+^ channels, or Kir (Hibino et al., 2010). Three Kir channel subfamilies are proposed to regulate neuronal excitability: Kir2 forms open or constitutively active inward rectifier K^+^ channels; Kir3 forms G protein-coupled inward rectifier K^+^ channels (GIRK) that are usually closed but in certain conditions can be open due to tonic activation by a G_i_/G_o_ protein-coupled receptor; Kir6 forms ATP-sensitive inward rectifier K^+^ channels in complexes with sulfonylurea receptors and are open when ATP levels are low (Stanfield et al., 2002; Hibino et al., 2010). All three Kir subfamilies are expressed in midline thalamus (Prüss et al., 2005; Thomzig et al., 2005; Saenz del Burgo et al., 2008). Recent observations using specific ion channel blockers suggest that all Kir channels are not only present in PVT neurons but also contribute to maintaining their RMPs (Hermes et al., 2013; Zhang et al., 2013). These findings are of particular interest since they indicate a role for GIRK channels in maintaining RMPs, a function previously detected in medial prefrontal cortex pyramidal (Witkowski et al., 2008) and locus ceruleus neurons (Torrecilla et al., 2002), but novel for thalamus.

### TASK-like channels

Resting K^+^ conductances are major contributors to neuronal RMPs, and their inhibition by neurotransmitters represents a key mechanism to modulate cell excitability (McCormick, 1992). Several members of the K^+^ two-pore-domain (K2P) channels are constitutively active at rest and exhibit properties expected of background “leak” channels (Goldstein et al., 2001; Patel and Honoré, 2001). Two of these, TWIK-related acid-sensitive K^+^ channel 1 (TASK-1) and TASK-3 channels, contribute substantially to the background membrane current in thalamocortical neurons (Meuth et al., 2006). TASK-1 and TASK-3 transcripts are also expressed in midline and intralaminar regions of thalamus (e.g., Talley et al., 2001) and observations in PVT neurons (Doroshenko and Renaud, 2009) also indicate the expression of a functional background pH-sensitive conductance with properties consistent with TASK-like channels. Briefly, PVT neurons demonstrate large shifts in RMP or membrane current (under voltage-clamp) in response to lowering or raising extracellular pH. These are due largely to changes in one or more K^+^ conductances that are reduced in acidic (pH 6.3) and enhanced in alkaline (pH 8.3) media. In addition, exposure to the local anesthetic bupivacaine or the endocannabinoid anandamide mimics the effects of acidic media by reducing K^+^ conductance, whereas exposure to the volatile anesthetic isoflurane induces membrane hyperpolarization by enhancing a K^+^ conductance (Maingret et al., 2001; Meuth et al., 2006; Veale et al., 2007).

### Slow afterhyperpolarizations (sAHPs)

The influx of Ca^2+^ ions through either HVA or LVA Ca^2+^ channels can have a variety of consequences, including the activation of different K^+^ conductances that contribute to membrane afterhyperpolarizations, or AHPs. AHPs provide neurons with an important intrinsic means of controlling their excitability and activity patterns over variable segments of time. AHPs are typically subdivided into three phases, fast (fAHP), medium (mAHP) and slow (sAHP), with different Ca^2+^-activated K^+^ channels (K_Ca_) contributing to each phase (Faber and Sah, 2003; Vogalis et al., 2003). PVT and other midline thalamic neurons express two prominent apamin-resistant sAHPs: a spike train-induced sAHP, and an LTS-induced sAHP (Figure 2D). Interestingly, neither sAHP appears to exist in neurons sampled in several other regions of thalamus (Figures 2E–G).

The ***spike train-induced sAHP*** in PVT neurons is abolished in the presence of TTX, suggesting that action potential-associated Ca^2+^ influx through HVA Ca^2+^ channels triggers sI_AHP_, the underlying current. An analysis of this sI_AHP_ (Zhang et al., 2010) identified the following characteristics: a dependency on Ca^2+^ influx; a contribution from each of the known HVA Ca^2+^ channel subtypes; a lack of sensitivity to known blockers of K_Ca_ channels; a significant reduction in the presence of a novel selective sAHP blocker UCL-2077 (Shah et al., 2006) and the nonselective K^+^ channel blockers barium and tetraethylammonium (TEA). In addition, blockade of HVA Ca^2+^ channels revealed an activity-dependent, Ca^2+^-independent component of the sAHP (see Figure 6 in Zhang et al., 2010) that exhibited the following properties: sensitivity to changes in [K^+^]_o_; insensitivity to changes in [Cl^−^]_i_; blockade by substitution of Na^+^ with Li^+^; sensitivity to quinidine (Zhang et al., 2010). These features are a hallmark of K_Na_ channels encoded by members of the *Slo* gene family, *Slo2.1* (Slick) and *Slo2.2* (Slack) that have a wide expression in brain, including PVT (Bhattacharjee et al., 2002, 2005; Bhattacharjee and Kaczmarek, 2005). Taken together, the data suggest that PVT neurons possess K_Ca_ channels that are principal contributors to the spike train-induced sAHPs at the lower end of the activity-dependent scale, together with K_Na_ channels that become progressively more engaged under conditions associated with more intense firing, as might occur during rhythmic bursting (Zhang et al., 2010).

The ***LTS-induced sAHP*** exhibits the following properties: long duration; an amplitude that is independent of the number of action potentials triggered by the LTS; resistance to TTX; dependence on Ca^2+^ influx and blockade by Ni^2+^; sensitivity to [K^+^]_o_; reduction by nonselective K^+^ channel blockers barium and TEA; insensitivity to specific K_Ca_ channel blockers (Zhang et al., 2009). In addition, in contrast to the spike train induced sAHPs, the LTS-induced sAHP lacks sensitivity to UCL-2077, suggesting involvement of different type(s) of K+ channel(s).

**Figure 3:**
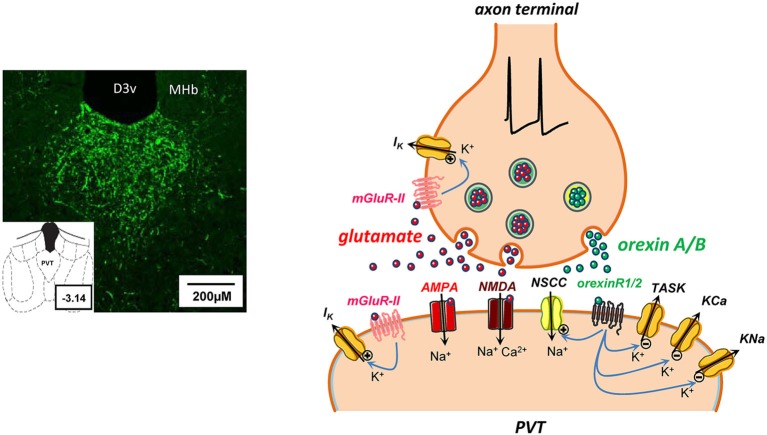
**Some potential consequences of glutamate and orexin co-release at a synapse in PVT**. On the left, microphotograph of coronal section from rat brain (bregma ∼ −3.14) reveal a dense distribution of orexin A-immunoreactive fibers in PVT nucleus. Abbreviations: D3v, dorsal 3rd ventricle; MHb, medial habenula. On the right, schematic synapse depicting action potential invasion of an axon terminal in PVT containing storage vesicles for a rapid transmitter (glutamate, red symbols) and a neuropeptide (orexin A or B, green symbols). Presynaptically released glutamate diffuses across the synaptic cleft to act at postsynaptic ionotropic AMPA and NMDA receptors, promoting cation influx and induction of rapid excitatory postsynaptic currents. In addition, glutamate release may potentially activate metabotropic group II (mGluR-II) receptors to open pre- and/or postsynaptic K^+^ channels (Hermes and Renaud, 2011). Activity-dependent co-release of orexins and activation of metabotropic orexin receptors (orexinR1/2) may have several postsynaptic actions that collectively result in enhanced neuronal excitability by: (a) opening of nonselective cation channels (NSCC; Kolaj et al., 2007); (b) closing of K^+^ channels, including two-pore-domain TASK-like channels that are constitutively active at rest (Doroshenko and Renaud, 2009); and (c) suppression of K_Ca_ and K_Na_ channels underlying spike train induced sAHPs (Zhang et al., 2010).

The observed prevalence of sAHPs in midline thalamus raises the notion that neurons in this part of thalamus are endowed with unique types of K^+^ channels. Functionally, the sAHP may be an important intrinsic mechanism governing typical rhythmic activities within this region of thalamus, and a possible target for neurotransmitter receptors (Zhang et al., 2009, 2010; see Orexins below, and Figure 3). Another functional consequence of large sAHPs is a propensity for spike frequency adaptation, a feature that exhibits variable expression in PVT neurons (Zhang et al., 2010).

## Neuropharmacology

### Amino acids

#### γ-aminobutyric acid (GABA)

GABA is the dominant inhibitory neurotransmitter in the forebrain, regulating cell excitability via rapidly acting anion-permeable ionotropic GABA_A_ receptors (GABA_A_Rs) and slowly acting, K^+^-permeable, G protein-coupled metabotropic GABA_B_ receptors (GABA_B_Rs). In the mammalian thalamus, GABAergic local circuit neurons have a differential and selective distribution in different species. In primates and carnivores, they are abundant in most of the dorsal thalamic nuclei. However, in rodents, they are found sparingly in intralaminar nuclei and are virtually absent in midline nuclei, including PVT (Arcelli et al., 1997). Nonetheless, the neuropil in rodent midline nuclei displays a high density of GABA-immunoreactive fibers and terminal boutons (Ottersen and Storm-Mathisen, 1984). In PVT, a portion of this input likely originates in the hypothalamic suprachiasmatic nuclei (SCN; Peng and Bentivoglio, 2004) where the majority of neurons express the GABA synthesizing enzyme glutamic acid decarboxylase (GAD; Moore and Speh, 1993; Kawano et al., 2001). Another potential source of GABAergic input to PVT originates from subpopulations of GAD-expressing arcuate nucleus neurons that co-express POMC or its posttranslational product β-endorphin (Bloom et al., 1978; Wittmann et al., 2013) or agouti-related protein (AgRP; Horvath et al., 1997; Cansell et al., 2012; Betley et al., 2013). The following pertains to GABA’s actions and potential roles in PVT.

**Ionotropic GABA_A_Rs** are composed of a combination of subunits (i.e., αβγδεθρ) that dictate the cellular distribution, physiological properties and pharmacology of the receptor. In ventrobasal (VB) thalamic nucleus neurons, *synaptically* located α1β2γ2-containing receptors mediate the “phasic” postsynaptic form of GABAergic inhibition, via receptors permeable to Cl^−^ and to a lesser extent $HCO_{3}^{-}$ (Farrant and Nusser, 2005). While details of GABA_A_R subunit composition remain to be defined in PVT neurons, these neurons clearly display “phasic” inhibition in the form of evoked, spontaneous, or miniature inhibitory postsynaptic currents (eIPSCs, sIPSCs, mIPSCs). An example is found in the analysis of the SCN innervation to anterior PVT (Zhang et al., 2006b). In brain slice preparations that maintain the integrity of the SCN innervation to PVT, electrical stimulation in SCN can evoke IPSCs that display a reversal potential around −70 mV, close to the calculated chloride ion equilibrium potential (~ −66 mV at 22°C) for this preparation (Zhang et al., 2006b). Such phasic IPSCs are reversibly suppressed by the GABA_A_R antagonists bicuculline and SR95531, and also by the GABA_A_R channel blocker picrotoxin. Many VB thalamic nucleus neurons also display *extrasynaptic* GABA_A_Rs that contain α4 and/or δ subunits which are known to mediate a “tonic” form of inhibition, initiated by ambient levels of extracellular GABA that escape presynaptic reuptake (Glykys and Mody, 2007; Belelli et al., 2009). Tonic GABA_A_Rs are “ON” all the time, and their sensitivity to neurosteroids, general anesthetics and alcohol make them potential therapeutic targets (Brinkley and Mody, 2012). While tonic GABA_A_Rs have not yet received detailed investigation in midline thalamus, a trademark of their presence in PVT neurons would be the detection of a steady outward membrane current in Cl^−^-loaded cells upon application of SR95531.

**Metabotropic GABA_B_Rs** operate via Gα_i/o_-type G proteins to regulate cell excitability through both postsynaptic and presynaptic modulation of K^+^ ion channels (Gassmann and Bettler, 2012). GABA_B_Rs are prominently expressed in the midline thalamus (Margeta-Mitrovic et al., 1999), yet there is little information on their neuropharmacology.

The available data imply that GABA_A_Rs and GABA_B_Rs are likely to have a role in the neurophysiology (and possibly neuropathology) in midline thalamus. In rodents, agonists targeting GABARs specifically in PVT induce major changes in behavior, including induction of feeding (Stratford and Wirtshafer, 2013) and abolition of cocaine-conditioned place preference (Browning et al., 2014). Studies in a rat model of chronic temporal lobe epilepsy (limbic epilepsy) reveal significant changes in eIPSCs and GABA_A_R subunit expression in PVT (Rajasekaran et al., 2009). One might speculate that changes in GABARs contribute to the exceptional sensitivity (i.e., *c-Fos* expression) of PVT neurons to various stressors. Interestingly, in rodents subjected to an acute behavioral stress, an evaluation of neurons in the hypothalamic parvicellular paraventricular nucleus reveals changes in their postsynaptic chloride ion gradients, rendering GABA_A_R signaling excitatory rather than inhibitory (Hewitt et al., 2009). Future investigations need to consider whether impairment in synaptic (phasic) or extrasynaptic (tonic) GABAR-mediated inhibition contributes to various CNS disorders where midline thalamus may be involved (see Brinkley and Mody, 2012). GABA_B_R ligands have already shown potential therapeutic value in various neurological (epilepsy) and neuropsychiatric disorders (e.g., schizophrenia and addictions; Bettler et al., 2004).

### Glutamate

Glutamate is recognized as the dominant CNS excitatory neurotransmitter interacting with ionotropic (AMPA, kainate (KA) and NMDA) receptors to mediate rapid excitatory transmission, and with G protein-coupled metabotropic glutamate receptors (mGluRs) to mediate slow excitatory or inhibitory actions (reviewed in Salt and Eaton, 1996). Both types of receptors are present in midline thalamus and PVT neurons exhibit properties consistent with actions mediated by both types of glutamate receptors (see below). Glutamatergic neurons can be identified by their co-expression of vesicular glutamate transporters (VGLUTs), considered definitive markers for neurons and axon terminals that synthesize and/or release glutamate (e.g., Fremeau et al., 2004). PVT neurons express VGLUT2 (Hur and Zaborszky, 2005) and (should they have local axon collaterals) may be an intranuclear source of glutamatergic input. VGLUTs serve to identify many sources of glutamatergic innervation to PVT, including: brainstem neurons containing markers for monamines and neuropeptides (Mestikawy et al., 2011; Schöne and Burdakov, 2012); lateral hypothalamic orexin/hypocretin neurons (Torrealba et al., 2003; Henny et al., 2010), recently shown with optogenetic probes to evoke typical rapid glutamatergic responses in neurons targeted by their axons (Schöne et al., 2012; Figure 3); a subpopulation of SCN neurons (Ziegler et al., 2002; Kiss et al., 2007), consistent with observations that electrical or chemical stimulation in SCN can evoke ionotropic AMPA-NMDA glutamate receptor-mediated excitation in PVT neurons (Zhang et al., 2006b); prefrontal and other corticothalamic inputs.

#### Ionotropic glutamate receptors (GluRs)

Following electrical stimulation in local or more distant sites (e.g., area of SCN), PVT neurons display evoked excitatory postsynaptic currents (eEPSCs) or potentials (eEPSPs) that contain components attributed to both AMPA/KA and NMDA ionotropic receptor subtypes (see Figure 3 in Zhang et al., 2006b). Depending on the MP, eEPSPs can trigger a single action potential or a LTS-induced burst of action potentials (see Figure 5 in Zhang et al., 2006b). PVT neurons also display abundant spontaneous AMPA/KA receptor-mediated excitatory postsynaptic potentials (sEPSPs) that can contribute to RMPs and therefore support ongoing burst firing (see Figure 4 in Hermes and Renaud, 2011). Curiously, voltage clamp recordings reveal that the equivalent AMPA/KA receptor-mediated EPSCs show little change in their frequency in the presence of TTX (see Figure 4 in Hermes and Renaud, 2011)), suggesting that glutamatergic axons terminating on PVT neurons may have unique properties that allow significant stochastic or spontaneous release of glutamate from nerve terminals.

***Metabotropic glutamate receptors (mGluRs)*** are a heterogeneous collection of C-type G protein-coupled receptors that are currently divided into three groups (I, II and III), each having a discrete CNS distribution (Niswender and Conn, 2010). Group II mGluRs are selectively found in limbic and forebrain regions, and their activation suppresses excitatory transmission in amygdala, bed nucleus of stria terminalis and prefrontal cortex (Lin et al., 2000; Marek et al., 2000; Grueter and Winder, 2005; Muly et al., 2007), sites known to receive a prominent innervation from PVT neurons. PVT neurons themselves also display a high expression of group II mGluRs (Ohishi et al., 1993; Gu et al., 2008) and a recent analysis (Hermes and Renaud, 2011) reveals that their postsynaptic activation by the selective group II mGluR orthosteric agonists LY379268 and DCG-IV induces membrane hyperpolarization, sufficient to suppress ongoing burst or tonic firing. In PVT, LY 379268 also activates presynaptic receptors to reduce ionotropic AMPA/KA receptor-mediated miniature excitatory postsynaptic currents (mEPSCs) arising from spontaneous synaptic release of glutamate. LY 487379, an mGluR2-positive allosteric modulator, potentiates both the postsynaptic and presynaptic actions of LY 379268 (Hermes and Renaud, 2011). These data support the notion that orthosteric activation or positive allosteric modulation of mGluR2s in PVT and midline thalamus may contribute to a reduction in excitatory neurotransmission to limbic and forebrain regions. This action may be a component of the central mechanisms underlying the beneficial effects of mGluR2-interacting drugs in animal models of anxiety and psychosis, disorders believed to result from aberrant or excessive glutamatergic neurotransmission (Swanson et al., 2005; Conn et al., 2009; Niswender and Conn, 2010).

### Monoamines

Many immunohistochemical studies reveal a prominent monoaminergic innervation to midline thalamus. PVT in particular receives noradrenergic fibers from locus coeruleus neurons, adrenergic and dopaminergic innervations from several brainstem and hypothalamic cell groups, and a serotonin innervation from the dorsal and median raphe nuclei (Lindvall et al., 1974; Swanson and Hartman, 1975; Cropper et al., 1984; Otake and Ruggerio, 1995). The midline and intralaminar thalamus is relatively rich in receptors and transporters for monoamines, however there is limited information on their presynaptic and/or postsynaptic localization or on their influence on membrane excitability and rapid neurotransmission. In the tree shrew, chronic psychosocial stress is reported to upregulate α2 receptors in PVT, and exposure of PVT neurons to noradrenaline elicits membrane depolarization mediated via postsynaptic α1 receptors and a GIRK conductance-mediated hyperpolarization via postsynaptic α2 receptors (Heilbronner et al., 2004; Heilbronner and Flügge, 2005).

Postsynaptic monoaminergic receptors can influence additional conductances beyond those that regulate MP. A notable example is their ability to modulate sAHPs and thereby change neuronal excitability (Haas and Konnerth, 1983; Madison and Nicoll, 1984; Pedarzani and Storm, 1993). This also applies to both types of sAHP that are observed in PVT neurons. The LTS-induced sAHP is significantly suppressed by isoproterenol, a β-adrenoceptor agonist, 5-CT, a serotonin 5HT_7_ receptor agonist, and by stimulation of the cAMP/protein kinase A signaling pathway (Zhang et al., 2009). In addition, Goaillard and Vincent (2002) reported suppression of the spike train-induced sAHP in midline and intralaminar thalamic neurons by a selective 5-HT_7_ agonist, an effect mediated by the cAMP messenger pathway. We recently reported that the spike train-induced sAHP in PVT neurons can be suppressed by transmitter molecules that engage either the cAMP or the PKC signaling cascade (Zhang et al., 2010).

### Neuropeptides

Various neuropeptide transmitters and their receptors have been reported in PVT (Tables 1 and 2), but relatively few have been evaluated for their influence on neuronal excitability.

**Table 1:** Summary table of electrophysiologically characterized postsynaptic neurotransmitter receptors in PVT neurons.

receptor type	effect(s)	References
bombesin - BB2	↓ Kir2 and ↓ TRPV1	Hermes et al., 2013
TRH	↓ GIRK and ↓ TRPC4/5	Zhang et al., 2013
orexin	↓ undefined K+ & ↓ undefined NSCC	Kolaj et al., 2007; Huang et al., 2006
	↓ undefined K+	Ishibashi et al., 2005
	↓ TASK	Doroshenko and Renaud, 2009
	↓ slow AHP (↓KCa & KNa)	Zhang et al., 2010
	↓ slow AHP (LTS-induced)	Zhang et al., 2009
vasopressin - V1a	↓ undefined K+	Zhang et al., 2006a
mGluR group II	↑ undefined K+	Hermes and Renaud, 2011
µ-opioid	↑ GIRK	Brunton and Charpak, 1998
α2 adrenoceptor	↑ GIRK	Heilbronner and Flugge, 2005
α1 adrenoceptor	↓ undefined K+	Heilbronner and Flugge, 2005
ß-adrenoceptor	↓ slow AHP (LTS-induced)	Zhang et al., 2009
5 HT7	↓ slow AHP (LTS-induced)	Zhang et al., 2009

**Table 2:** Summary table of additional neurotransmitter G-protein coupled receptors expressed in PVT neurons. Most data refer to receptor mRNA analyses. Receptor densities are directly copied from actual references.

receptor type		density	species	references
VIP	VPAC1	2/5	rat	Joo et al., 2004
	VPAC2	4/5		
	PAC1	0/5		
neuropeptide Y	Y1	3/3	rat	Parker and Herzog, 1999
	Y2	1/3		
	Y4	0/3		
	Y5	2/3		
histamine	H1	3/4	human, rat	Jin et al., 2002 and 2005
	H2	1/4		
	H3	3/4		
neuropeptide S	NPS	2/4	rat	Xu et al., 2007
prokineticin	PK1	0/4	mouse	Cheng et al., 2006
	PK2	4/4		
glucocorticoid	GR	2/3	rat	Morimoto et al., 1996
estrogen	beta	2/3	mouse	Mitra et al., 2003
CRH	CRF1	3/4	mouse	Chen et al., 2000
melanocortin	MCHR	2/4	rat	Saito et al., 2001
adenosine	A1	4/4	rat	Ochiishi et al., 1999
oxytocin	OxR	2/4	rat	Yoshimura et al., 1993
opioids	mu	2/3	rat	Ding et al., 1996
	delta	0/3		George et al., 1994
	kappa	3/3		
GABAb	GABAbR1	3/5	rat	Margeta-Mitrovic et al., 1999
metabotropic	mGluR1	2/3	rat	Neto et al., 2000
glutamate	mGluR5	1/3		
	mGluR4	2/3		
	mGluR7	1/3		
cholecystokinin	CCKB	present	rat	Bhatnagar et al., 2000
substance P	NK1	4/4	monkey	Rigby et al., 2005
	NK2	0/4		
	NK3	1/4		
dopamine	D2/3	high	human	Rieck et al., 2004
leptin	LepRb	1/4	mouse	Scott et al., 2009
cannabinoid	CB1	1/4	rat	Jelsing et al., 2008
	CB2	light	rat	Gong et al., 2006
muscarinic	M1	2/3	rat	Mash and Potter, 1986
	M2	2/3	rat	
	M3	moderate	rat	Levey et al., 1994

Most data refer to receptor mRNA analyses. Receptor densities are directly copied from actual references.

#### Arginine vasopressin (AVP)

Fibers displaying AVP-like immunoreactivity are present throughout the rostrocaudal extent of PVT, and arise almost exclusively from AVP synthesizing neurons located in the dorsomedial part of the SCN (Buijs et al., 1978; Sofroniew and Weindl, 1978; Watts and Swanson, 1987). PVT neurons exposed to bath-applied AVP respond with a TTX-resistant membrane depolarization, blockable by prior application of a selective V1a receptor antagonist, and not replicated by a specific V2 receptor type agonist (Zhang et al., 2006a). The AVP-induced response is associated with reduction in an inward rectifier K^+^ conductance. In addition, after correction of the AVP induced membrane depolarization through intracellular injection of negative current, an increase in the LTS duration and number of superimposed Na^+^ spikes may reflect a change in an LTS-associated nonselective cation conductance (see Figure 3 in Zhang et al., 2006a). Interestingly these AVP-induced effects are more prevalent in neurons recorded from posterior PVT suggesting heterogeneity along the rostral-caudal axis. Since AVP release from SCN provides an important circadian output signal for regulating neuroendocrine rhythms (see Kalsbeek et al., 2010 for review), its release from synaptic terminals in PVT may serve to mediate rhythmic information to the limbic system.

#### Gastrin-releasing peptide (GRP)

GRP, a 27-amino acid peptide, and the decapeptide neuromedin B (NMB) are mammalian analogues of the amphibian bombesin (BB) and BB-like peptides present in brain (Jensen et al., 2008). Central GRP and its receptor have been implicated in a variety of functions, notably feeding, circadian rhythms, emotion, fear-related memory processing, itch sensation and sexual behavior (Moody and Merali, 2004; Sun and Chen, 2007; Sakamoto et al., 2008). In addition, GRP receptor expression and signaling may have a role in CNS disorders that include anxiety, autism, memory dysfunction and brain tumors (reviewed in Roesler and Schwartsmann, 2012). Initial studies using autoradiography revealed a high density of BB binding in PVT and in centromedial, paracentral and other intralaminar thalamic nuclei (Zarbin et al., 1985). With immunocytochemistry, fibers displaying GRP-like immunoreactivity have also been reported in PVT and select midline and intralaminar nuclei (Mikkelsen et al., 1991; Hermes et al., 2013). A recent electrophysiological investigation (Hermes et al., 2013) revealed that >90% of PVT neurons exposed to nanomolar concentrations of GRP respond with a TTX-resistant postsynaptic membrane depolarization. GRP-induced depolarizations appear to be associated with two patterns of firing: one population of PVT neurons with relatively hyperpolarized RMPs responded with sequences of LTS-mediated bursts of action potentials recurring at low frequencies; another population of PVT neurons with more depolarized RMPs responded with tonic firing. GRP’s effects in PVT are mediated selectively by postsynaptic BB type 2 (BB_2_) receptors, consistent with the nanomolar affinity of BB_2_ receptors for GRP (Jensen et al., 2008). While these findings contrast with those from earlier investigations that reported an absence of BB_2_ receptors in midline and intralaminar thalamus (Ladenheim et al., 1992; Wada et al., 1992), more recent data obtained in mouse brain^1^ support the existence of BB_2_ receptors in these regions of the thalamus.

Voltage-clamp analysis suggests that GRP’s activation of PVT neurons involves two ionic mechanisms: suppression of a Ba^2+^-sensitive, presumably Kir2 type, inward rectifier K^+^ conductance, and concomitant activation of a nonselective cation conductance with a transient receptor potential (TRP) vanilloid 1 (TRPV1)-like pharmacological profile (Hermes et al., 2013). TRPV1-expression has been reported, and disputed, in midline and intralaminar thalamus (Mezey et al., 2000; Roberts et al., 2004; Cavanaugh et al., 2011). Importantly, application of a selective TRPV1 antagonist (SB 366791) can be seen to reduce GRP-induced membrane depolarization and rhythmic burst or tonic firing, implying that activation of a TRPV1-like conductance does indeed contribute significantly to GRP-induced increases in PVT neuronal excitability. We (Hermes et al., 2013) have cautiously applied the term “TRPV1-like” to this action since some biophysical properties differ from previously reported TRPV1 features, possibly because TRPV1 forms heteromers with other TRPV channel subunits or members of a different TRP family, or is a splice variant of TRPV1 that has different functional properties. The origin of GRP immunoreactive fibers in midline thalamus likely includes a subpopulation of SCN neurons where GRP is a co-existing neuropeptide (van den Pol and Tsujimoto, 1985), raising speculation that the described membrane actions could in part mediate circadian-related information to the limbic system.

#### Orexins (Hypocretins)

Orexin A (hypocretinA) and orexin B (hypocretinB) are neuropeptides synthesized by a select population of lateral hypothalamic-perifornical neurons whose axons display a wide but selective CNS distribution (de Lecea et al., 1998; Sakurai et al., 1998). Two G protein-coupled orexin receptors, OXR1 and OXR2, have been characterized and their distribution mapped (Marcus et al., 2001). Since their discovery, orexins have been shown to participate in multiple physiological functions, including vigilance and arousal, feeding and energy homeostasis, reward-motivated behaviors and stress responses (reviewed in Kukkonen, 2013). Electrophysiological data from neurons in different brain regions verify that activation of orexin receptors potently enhances neuronal excitability (Kukkonen, 2013). It is notable that the midline thalamus, and PVT in particular, receives one of the most dense orexinergic innervations in the CNS (Peyron et al., 1998; Figure 3). PVT contains both OXR1 and OXR2 (Marcus et al., 2001). Consistent with this distribution of orexin fibers and receptor are electrophysiological findings that subpopulations of neurons in the midline thalamic nuclei, but not in ventrolateral thalamus, respond to both orexin A and orexin B (Bayer et al., 2002). Responsive neurons exhibit membrane depolarization when exposed to low nanomolar concentrations of orexins, with orexin B appearing more potent than orexin A in reducing a postsynaptic membrane K^+^ conductance (Bayer et al., 2002; Ishibashi et al., 2005; Govindaiah and Cox, 2006; Huang et al., 2006; Kolaj et al., 2007). Our observations in PVT neurons exposed to high nanomolar concentrations of either orexin A or orexin B indicate that orexin receptor activation results in reduction in one or more K^+^ conductances that include TASK-like channels, and increase in a nonselective cation conductance (Kolaj et al., 2007; Doroshenko and Renaud, 2009). Orexin-induced responses depend on both the cell’s MP and the amplitude of the induced depolarization to achieve either burst or tonic firing; an associated increase in the LTS duration promotes a significant increase in the number of superimposed action potentials (see Figure 4 in Kolaj et al., 2007). We recently reported that orexin A also suppressed the Ca^2+^-dependent (K_Ca_) and Na^+^-dependent (K_Na_) components of the spike train-induced sAHP (Zhang et al., 2009) and the LTS-induced sAHP (Zhang et al., 2010). Thus, as schematically shown in Figure 3, suppression of sAHPs adds a novel dimension to the manner whereby orexins can enhance excitability in PVT neurons, potentially expanding the response elicited by release of glutamate, a co-existing transmitter in orexin-synthesizing neurons (see Schöne et al., 2012). Of note, it has recently been proposed that OXR1 and OXR2 might engage different signaling pathways and thus may have distinctly different actions (e.g., Mieda et al., 2013; Xu et al., 2013). No doubt the availability of receptor selective antagonists (Lebold et al., 2013) will facilitate further investigation into potential differential actions of these peptides (e.g., Xu et al., 2013).

#### Thyrotropin releasing hormone (TRH)

TRH, the first hypothalamic releasing factor to be isolated and characterized in the late 1960s, is present in multiple extrahypothalamic brain areas where it has a role in arousal, mood and cognition (see reviews by Lechan and Fekete, 2006; Yarbrough et al., 2007). TRH and TRH analogs are also being evaluated for their therapeutic potential in areas of neuroprotection, psychiatric and mood disorders, narcolepsy and certain forms of epilepsy (Gary et al., 2003; Yarbrough et al., 2007). Among CNS sites displaying a high density of TRH binding and mRNA expression for TRH receptors is the midline thalamus, with PVT exhibiting mRNA for both presently known receptors, TRHR1 and TRHR2 (Heuer et al., 2000). Since little is known of its actions at the cellular level in midline thalamus, we recently explored the response of PVT and neighboring central medial thalamic neurons to exogenous application of TRH and a TRH analog, taltirelin (Zhang et al., 2013). The data indicated that >90% of PVT neurons responded to nanomolar concentrations of TRH, and taltirelin, with a desensitizing G protein-mediated membrane depolarization and inward current that involved suppression of a GIRK-like inward rectifier K^+^ conductance and activation of a transient receptor potential, canonical (TRPC)-like nonselective cation conductance, possibly involving TRPC4/C5 subunits. Similar to observations with AVP, orexins and GRP, the enhanced excitability of PVT neurons to TRH also included a marked enhancement of LTS-evoked firing, a feature that was significantly attenuated in the presence of putative TRPC blockers. Although, no specific TRH receptor antagonists have yet been identified, pretreatment with the benzodiazepine chlordiazepoxide, considered as a competitive TRH receptor antagonist in the adenohypophysis where only TRHR1 is present (Sun et al., 2003), significantly reduced the TRH-induced inward current in PVT. In addition, in neighboring central medial nucleus, where only TRHR2 mRNA has been reported (Heuer et al., 2000), a response to TRH required significantly higher concentrations of the peptide and lacked sensitivity to chlordiazepoxide, supporting the notion that TRHR1 mediates the response to TRH in PVT neurons. TRH-immunoreactive axons detected in PVT originate in part from TRH synthesizing neurons in hypothalamus and brainstem (Merchenthaler et al., 1988; Wittmann et al., 2009), suggesting that TRH may contribute in the integration of the metabolic and energy sensing functions of the hypothalamus with motivated behavior functions attributed to midline thalamus.

## Summary and perspectives

A growing body of literature attests to the involvement of midline and intralaminar thalamic neurons in various distinct functions and behaviors, including vigilance and arousal, nociception, stress responses and motivated behaviors. Comparatively little is known about the intrinsic cellular properties and neuropharmacology of neurons in this area of CNS, fundamental for a full understanding of midline and intralaminar thalamic function. We have reviewed present knowledge on the cellular physiology and pharmacology of neurons in PVT, a stable midline thalamic structure throughout mammalian evolution. PVT appears to be a nodal point in CNS circuits related to salt appetite, energy balance and food reward, and where neurons display exceptional sensitivity (early gene expression) to stress and arousal. Data derived from patch clamp recordings in brain slices provide insights on properties of PVT neurons, some of which are not detectable in neurons sampled in VB and reticular thalamic nuclei.

RMP of PVT neurons is maintained by K^+^ channels, including inward rectifier (Kir) and two pore domain (TASK-like) channels, and by HCN channels. These channels also participate in a diurnal fluctuation in RMP of PVT neurons, with an additional change in properties of LVA T-type Ca^2+^ channels contributing to an increase in spontaneous burst firing during the night period, when rodents are most active. The mechanisms underlying these diurnal changes remain undefined, but are likely to involve an influence of SCN circadian pacemaker neurons which directly innervate PVT neurons.

PVT and a sample of midline thalamic neurons display two types of apamin-resistant sAHPs that may prove to be a defining characteristic of neurons in this area of brain, given that they are not seen in neurons sampled in thalamic VB or reticular nuclei. A TTX-sensitive spike train-induced sAHP is produced by K_Ca_ channels with unusual pharmacological characteristics and, at higher levels of activity, by K_Na_ channels. Another is a TTX-resistant LTS-induced sAHP that is mediated by K^+^ channels insensitive to known specific K_Ca_ channel blockers. Both types of sAHP are subject to modulation by activation of neurotransmitter receptors, suggesting that they may play an important role in regulating neuronal excitability in the midline (and possibly intralaminar) thalamus.

A comparison of receptors currently under investigation in PVT (Table 1) with all the receptors presently known to be expressed in PVT (Table 2) indicates that we are in the early stages of understanding the properties and neuropharmacology of PVT neurons. Investigations to date (Table 1) have established mainly the postsynaptic actions mediated by specific transmitter receptors for GABA, glutamate, several monoamines and peptide neurotransmitters, with more to come. Whereas the focus here has been on PVT, the intrinsic properties and neuropharmacology of neurons in other midline and intralaminar nuclei also need to be characterized. These initial observations attest to some fundamental functional differences between midline and lateral/reticular thalamic neurons. Since specific regions of the midline and intralaminar thalamus can be associated with specific behaviors, there is a high probability of detecting different characteristics at the cellular level. Thus, this review represents a work in progress, with the prospects that the introduction of newer technologies (e.g., optogenetics) and animal models to *in-vivo* and *in-vitro* investigations will bring us to a better understanding of this relatively unexplored region of the mammalian thalamus.

## Conflict of interest statement

The authors declare that the research was conducted in the absence of any commercial or financial relationship that could be construed as a potential conflict of interest.

## Footnotes

^1^http://www.alleninstitute.org
